# Metabolically Protective Cytokines Adiponectin and Fibroblast Growth Factor-21 Are Increased by Acute Overfeeding in Healthy Humans

**DOI:** 10.1371/journal.pone.0078864

**Published:** 2013-10-18

**Authors:** Leonie K. Heilbronn, Lesley V. Campbell, Aimin Xu, Dorit Samocha-Bonet

**Affiliations:** 1 Discipline of Medicine, University of Adelaide, Adelaide, South Australia, Australia; 2 Research Center for Reproductive Health, University of Adelaide, Adelaide, South Australia, Australia; 3 Garvan Institute of Medical Research, Sydney, New South Wales, Australia; 4 Faculty of Medicine, University of New South Wales, Sydney, New South Wales, Australia; 5 Department of Medicine, Department of Pharmacology and Pharmacy, and Research Center of Heart, Brain, Hormone and Healthy Ageing, University of Hong Kong, Hong Kong; Pontificia Universidad Catolica de Chile, Chile

## Abstract

**Context:**

Circulating levels of metabolically protective and adverse cytokines are altered in obese humans and rodent models. However, it is not clear whether these cytokines are altered rapidly in response to over-nutrition, or as a later consequence of the obese state.

**Methods:**

Forty sedentary healthy individuals were examined prior to and at 3 and 28 days of high fat overfeeding (+1250 kCal/day, 45% fat). Insulin sensitivity (hyperinsulinaemic-euglycaemic clamp), adiposity, serum levels of adiponectin and fibroblast growth factor-21 (FGF21), fatty acid binding protein-4 (FABP4), lipocalin-2 and plasminogen activator factor-1 (PAI1) were assessed. Statistics were performed by repeated measures ANOVA.

**Results:**

Overfeeding increased weight, body fat and liver fat, fasting glucose, insulin and reduced insulin sensitivity by clamp (all P <0.05). Metabolically protective cytokines, adiponectin and FGF21 were increased at day 3 of overfeeding (P ≤0.001) and adiponectin was also elevated at day 28 (P=0.001). FABP4, lipocalin-2 and PAI-1 were not changed by overfeeding at either time point.

**Conclusion:**

Metabolically protective cytokines, adiponectin and FGF-21, were increased by over nutrition and weight gain in healthy humans, despite increases in insulin resistance. We speculate that this was in attempt to maintain glucose homeostasis in a state of nutritional excess. PAI-I, FABP4 and lipocalin 2 were not altered by overfeeding suggesting that changes in these cytokines may be a later consequence of the obese state.

**Clinical trial registration:** www.clinicaltrials.gov (NCT00562393)

## Introduction

Obesity is associated with increased secretion of metabolically adverse adipokines such as fatty acid binding protein-4 (FABP4) and reduced secretion of metabolically protective adipokines such as adiponectin [1]. Obesity is also associated with ectopic lipid deposition in the liver [2] and altered secretion of cytokines from the liver. Whilst the exact target and function of many novel cytokines remain to be elucidated, there is increasing evidence to suggest direct involvement in the pathogenesis of insulin resistance [3]. 

Adiponectin is an abundantly produced adipokine that is inversely related to adiposity, and exerts protective anti-inflammatory and anti-apoptotic effects that improve insulin sensitivity and reduce oxidative stress [4]. FABP4 is a lipid binding chaperone that interacts with hormone sensitive lipase in adipose tissue to facilitate lipolysis [5]. FABP4 is also released from adipocytes [6], although the mechanism, target and function of the secreted form of FABP4 is not clear. Circulating levels of FABP4 are positively correlated with obesity and non-alcoholic fatty liver [7] and predict the development of metabolic syndrome and type 2 diabetes [8]. Moreover, FABP4 disruption decreases insulin resistance, dyslipidemia and liver steatosis in obese or fat fed animals [9]. Lipocalin-2 was originally identified in human neutrophils, but is also secreted from adipose tissue, macrophages, liver, lung, and kidney and is involved in the inflammatory response [10]. Lipocalin-2 is elevated in obesity, diabetes and non-alcoholic fatty liver disease [7,11]. Plasminogen activator inhibitor-1 (PAI-1) is expressed in many tissues including visceral and subcutaneous adipose tissue depots, although adipose tissue-resident macrophages may be the primary source of this cytokine in these depots [12]. PAI-1 is the prime regulator of fibrinolysis and subjects with increased levels of PAI-1 are predisposed to atherothrombotic events [12]. Obese patients with and without type 2 diabetes have elevated levels of PAI-1 as compared to lean individuals and this is closely linked with insulin resistance [13]. 

Like adiponectin, the recently discovered and predominately liver derived cytokine, fibroblast growth factor-21 (FGF21) is considered to be metabolically protective. Over-expression or treatment with recombinant FGF21 protects mice from development of obesity and fatty liver and improves insulin sensitivity [3] and comparable metabolic benefits are also observed in rhesus monkeys [14]. Paradoxically, FGF21 levels are elevated in obesity [15] and obese mice treated with recombinant FGF21 have a blunted metabolic response to FGF21, which suggests that resistance to the actions of FGF21 may develop in obesity [16]. 

Short term overfeeding provides a model to examine whether cytokines are altered rapidly in response to over-nutrition. In this cohort, we have previously reported that short term overfeeding does not alter macrophage infiltration of adipose tissue, although increases in C-reactive protein (CRP) and macrophage chemo-attractant protein-1 (MCP1) were identified [17]. Here, we examined the relationships between adiposity and insulin resistance and circulating levels of adiponectin, FABP4, lipocalin-2, PAI-1 and FGF21 before and after high fat overfeeding for 28 days. 

## Methods

### Subjects and Study Design

The study was approved by the Human Research and Ethics Committee of St Vincent’s Hospital and registered as a Clinical Trial at clinicaltrials.gov, registration number NCT00562393, all participants signed informed consent prior to the study. The study protocol and CONSORT diagram is described previously [18,19] and this analysis includes the entire cohort as previously reported. 

The cohort consisted of 20 women (5 post-menopausal) and 20 men, mean age 37 years (range 21-59 years); 17 subjects reported a family history of type 2 diabetes. All subjects were healthy, sedentary and not taking any medications. All foods were provided for 3-days before metabolic testing at baseline, day 3 and study end as described previously [18]. At baseline, this was at calculated energy requirements, with a nutrient composition of 30% fat, 15% protein and 55% carbohydrate. During the first and last 3 days of overfeeding, all foods were provided at 1250 kcal/day above calculated energy requirements, with a nutrient composition of 45% fat, 15% protein and 40% carbohydrate. During the remainder of the overfeeding phase (days 3 - 25), subjects were asked to consume their regular diet with high fat snacks provided to increase energy intake by 1250 kcal/day. Average (± SEM) reported energy intakes during the self-selecting phase increased from 1980 ± 110 to 3100 ± 140 kcal/d and the contribution of fat from 34 ± 1 at baseline to 45 ± 1 %. Weight gain and compliance were monitored weekly by the research nurse and dietician. 

### Metabolic Testing at Baseline and after 28 Days of Overfeeding

All metabolic tests were conducted at the Clinical Research Facility at the Garvan Institute of Medical Research after a 12h overnight fast. Weight was measured in a hospital gown after voiding. Height, blood pressure, waist and hip circumference were also measured. Fasting blood samples were drawn and insulin sensitivity was assessed by a 2-h hyperinsulinaemic-euglycaemic clamp (60 mU/m^2^/min), as described [18]. Steady state glucose infusion rate (GIR) between 90 and 120 min of the clamp procedure was averaged and normalized for body fat free mass (FFM). Resting metabolic rate and respiratory quotient were assessed by True One metabolic cart following 30 minutes of supine rest (ParvoMedics, UT, USA). Body composition was measured by dual energy x-ray absorptiometry (DXA; Lunar DPX GE Lunar, Lunar Corp., Madison, WI). Three cross-sectional computed tomography (CT) scans (Phillips Gemini GXL), 1 cm-width, centered on the L2-L3 and L4-L5 disc space, and the T12-L1 disc space were also performed to assess abdominal adipose tissue distribution and hepatic fat content as described [18]. An additional blood sample for fasting levels of glucose, insulin and cytokines was taken at 3 days of overfeeding. 

### Biochemical variables

Blood glucose was determined by the glucose oxidase method (Glucose analyzer 2300 STAT PLUS 230V, YSI, Inc., Yellow Springs, OH, USA), serum insulin by radioimmunoassay (Linco, St. Charles, MO, USA) and serum non-esterified fatty acid (NEFA) by enzymatic colorimetric assay (Wako, Osaka, Japan). Adiponectin, lipocalin-2, PAI-1, FGF-21, and FABP4 were determined by immunoassays established by Antibody and Immunoassay Services, the University of Hong Kong [20-23].. The observed intra and inter assay CVs were 3.2% and 4.6%, 4.3% and 6.2%, 3.7% and 5.8%, 5.2% and 6.7%, and 4.5% and 6.3% respectively.

### Statistical Analysis

Data are presented as mean ± SEM unless otherwise stated. FABP4 and insulin data were log_10_-transformed prior to statistical analysis. Since no differences were detected for any markers of inflammation at baseline or in response to overfeeding between individuals with and without a family history of type 2 diabetes, these groups were combined. The effects of overfeeding were tested using repeated measures-ANOVA with gender as the between-subject factor. In case of significance a paried t test was used to evaluate differences between time-points. Pearson’s correlations were performed and partial correlations were used to adjust for gender. Sample size requirements were calculated for primary endpoint ∆ (glucose infusion rate/kg FFM) using 2 sided within subject contrasts with α<0.05 and statistical power 1-β>0.8. Statistical Package for Social Sciences (SPSS) version 21 (Chicago, IL) was used, without adjustment for multiplicity.

## Results

### Cytokine levels by gender and relationships with adiposity

**Figure 1:**
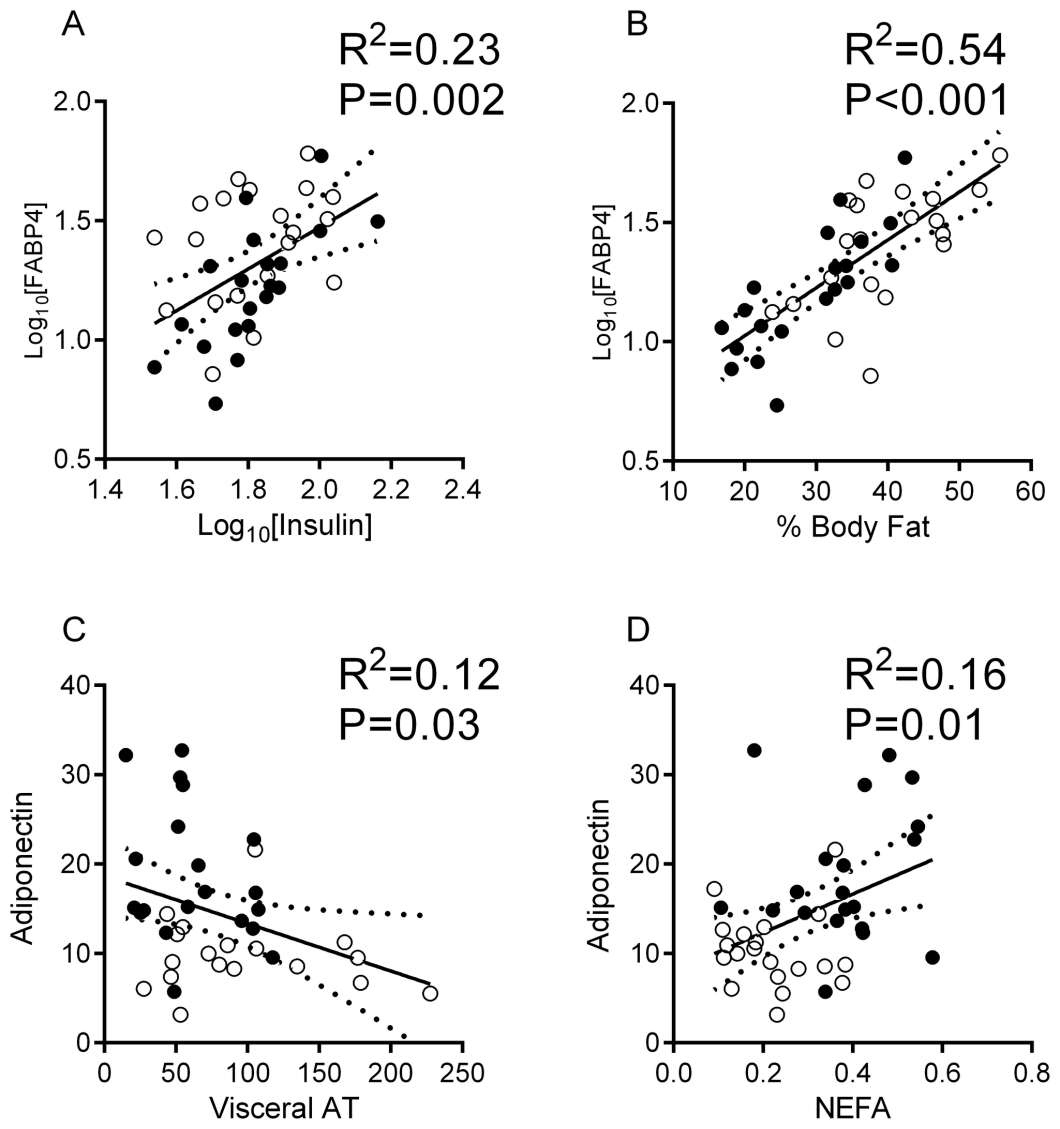
Relationships between circulating fatty acid binding protein 4 and fasting insulin (A) and percent body fat by DXA (B), and between adiponectin and visceral adipose tissue (C) and fasting serum non-esterified fatty acid (D) in men (empty circles) and women (dark circles). Depicted are the line of fit and the 95% confidence curves that were obtained from linear regression. Abbreviations: FABP4, fatty acid binding protein 4; AT, adipose tissue; NEFA, non-esterified fatty acid.

At baseline, significantly higher serum concentrations of FABP4 and adiponectin, and lower serum concentrations of FGF21 were observed in women (Table 1). FABP4 was closely correlated with fasting insulin (Figure 1A), body fat (Figure 1B), BMI (r^2^=0.22, P=0.002), abdominal subcutaneous (r^2^=0.4, P <0.001) and visceral adipose tissue (r^2^=0.15, P=0.02) and serum NEFA (r^2^=0.27, P <0.001). These associations remained significant following adjustment for gender (all P ≤0.02). Serum adiponectin was inversely correlated with visceral adipose tissue (Figure 1C) and positively with fasting NEFA (Figure 1D), although significance was lost after adjusting for gender. We did not detect any relationships between lipocalin-2, PAI-1, or FGF-21 and measures of adiposity, NEFA or insulin sensitivity, although a positive relationship was observed between FGF21 and the respiratory quotient (r^2^=0.26, P=0.002), which remained significant after adjusting for gender (P=0.008). 

**Table 1:** Gender differences in cytokine levels at baseline.

	Men	Women	P-Value
**FABP4 (µg/L)**	19.6 ± 2.9	28.9 ± 3.1	0.02
**Adiponectin (mg/L)**	10.3 ± 0.9	18.7 ± 1.2	<0.001
**FGF21 (ng/L)**	81.8 ± 13.5	48.3 ± 8.5	0.04
**Lipocalin-2 (µg/L)**	30.3 ± 4.0	27.9 ± 2.7	0.6
**PAI-1 (µg/L)**	6.0 ± 0.6	5.9 ± 0.6	0.9

Data are mean ± SEM

FABP4 data were log_10_-transformed prior to statistical analysis

### Metabolic consequences of overfeeding

**Figure 2:**
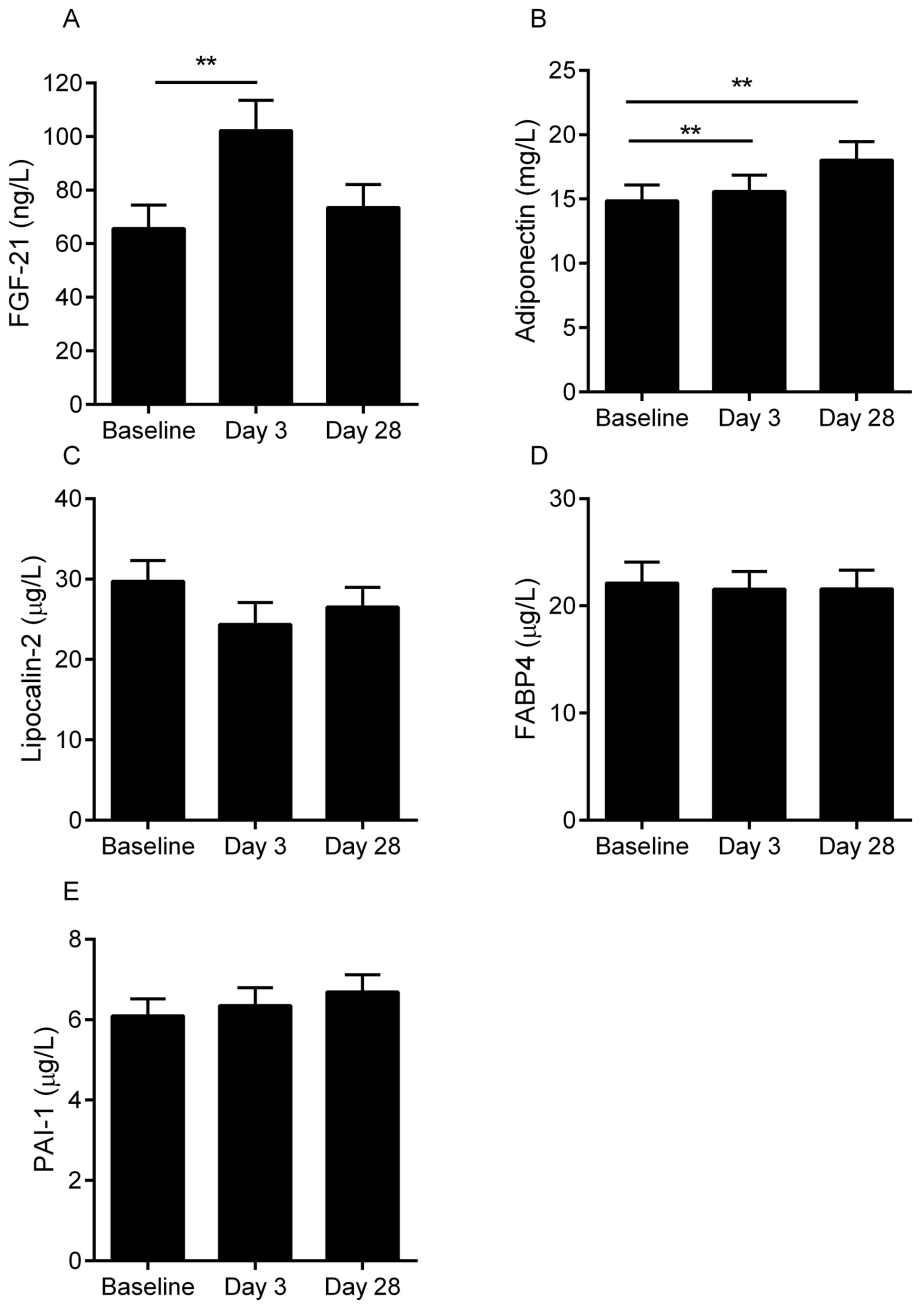
Serum concentrations of fibroblast growth factor 21 (A), adiponectin (B), lipocalin-2 (C), fatty acid binding protein 4 (D) and plasminogen activating inhibitor 1 (E) at baseline and in response to overfeeding in healthy humans. ^*^P <0.05, ^**^P ≤0.001. FABP4 data were log_10_-transformed prior to statistical analysis.

Anthropometric and metabolic measures at baseline and after 28 days of overfeeding are summarized in Table 2. Overfeeding induced significant weight gain (2.7 ± 0.3 kg, P <0.001). Similarly, BMI, percent body fat, abdominal subcutaneous and visceral adipose tissue and liver fat were significantly increased by overfeeding (Table 2, all P <0.01). Fasting glucose and insulin increased at both 3 and 28 days (P <0.01) and fasting NEFA decreased transiently at day 3 (P<0.001) and returned to baseline levels at day 28 (P=0.4). Insulin sensitivity by hyperinsulinemic clamp and HOMA-IR was reduced (P=0.03 and P<0.001, respectively). Resting metabolic rate (RMR) increased significantly (P≤0.003) and the respiratory quotient (RQ) increased significantly at day 3 (P<0.001) and returned to baseline levels on day 28 (P=0.2). Overfeeding transiently increased serum levels of FGF21 at day 3 (P=0.001, Figure 2A) and increased adiponectin levels at day 3 and day 28 (P ≤0.001, Figure 2B). The responses in serum adiponectin were independent of gender (P >0.2 for both), but FGF21 was increased to a greater extent in men at day 3 (P=0.04). No statistically significant changes were detected in lipocalin-2 (P=0.1, Figure 2C), FABP4 (P=0.7, Figure 2D) and PAI-1 (P=0.4, Figure 2E). 

**Table 2:** Anthropometric and metabolic responses to overfeeding.

		Overfeeding	
	**Baseline**	**3-days**	**28-days**
Weight (kg)	75.3±1.9	75.9±1.9^**^	78.1±1.9^**^
BMI (kg/m^2^)	25.6±0.6	25.8±3.6^**^	26.6±3.6^**^
Total body fat (%)	34±1	NA	35±1^**^
Abdominal subcutaneous fat (cm^2^)	256 ± 17	NA	276 ± 17^**^
Abdominal visceral fat (cm^2^)	77 ± 8	NA	86 ± 8^**^
Liver density (HU)^#^	55 ± 2	NA	53 ± 2^**^
Glucose (mmol/L)	4.5±0.06	4.7±0.06^**^	4.6±0.05^*^
Fasting insulin (pmol/L)	65.5±3.3	80.3±4.5^**^	77.2±3.6^**^
Fasting NEFA (mmol/L)	0.30 ± 0.02	0.19 ± 0.02^**^	0.30 ± 0.02
HOMA-IR	1.8±0.1	2.3±0.1^**^	2.2±0.1^**^
GIR (µmol/KgFFM/min)	54.8±2.8	NA	50.3±2.5^*^
Resting metabolic rate (kcal/day)	1378 ± 38	1454 ± 42^**^	1452 ± 42^**^
Respiratory quotient	0.81 ± 0.01	0.85 ± 0.01^**^	0.82 ± 0.01

Data are mean ± SEM

Difference from baseline ^*^P <0.05, ^**^P <0.01

^#^Liver density in Houndsfeld Units (HU) is inversely proportional to liver fat, (NA) not assessed at day 3.

Insulin data were log_10_-transformed prior to statistical analysis

Abbreviations: BMI, body mass index; NEFA, non-esterified fatty acids; HOMA-IR, homeostasis model assessment of insulin resistance; GIR, glucose infusion rate; FFM, fat free mass.

**Figure 3:**
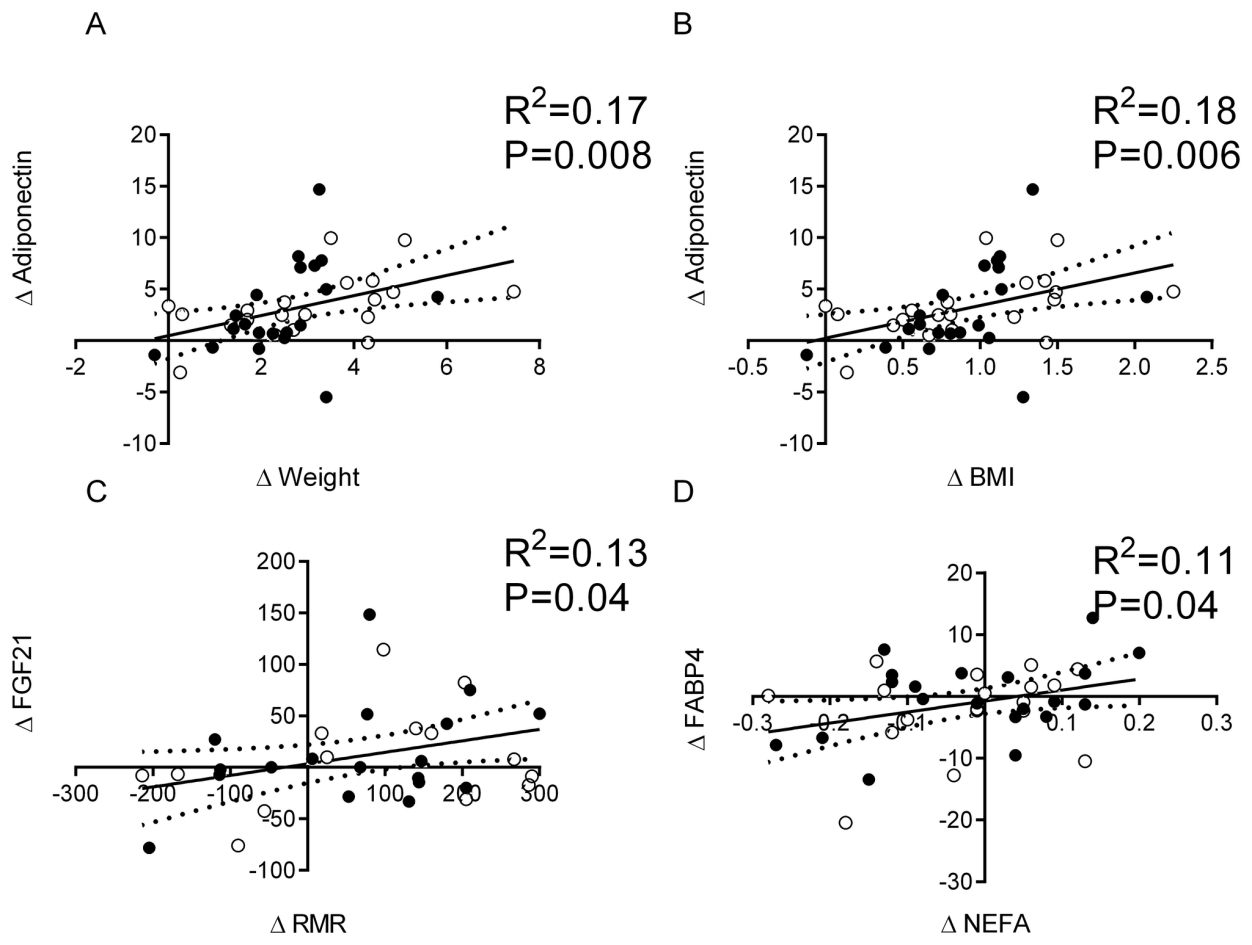
Associations between the change in adiponectin and change in weight (A) and BMI (B) and between the change in fatty acid binding protein 4 and change in fasting non-esterified fatty acid (C) and between the change in fibroblast growth factor 21 and resting metabolic rate (D) with overfeeding in men (empty circles) and women (dark circles). Change was calculated as day 28 minus baseline (∆). Depicted are the line of fit and the 95% confidence curves that were obtained from linear regression. Abbreviations: BMI, body mass index; FABP4, fatty acid binding protein 4; FGF21, fibroblast growth factor 21; RMR, resting metabolic rate.

Of the cytokines examined, only the change in adiponectin was correlated with weight gain and change in BMI at day 28 (Figure 3A and B, respectively). The change in FGF21 was positively related to the change in resting energy expenditure (Figure 3C) and the change in FABP4 correlated with the change in NEFA (Figure 3D). The change in PAI-1 and lipocalin-2 were not correlated with changes in any metabolic variable measured in response to overfeeding.

## Discussion

The secretion profile of pro and anti-inflammatory cytokines is altered in obesity and may directly impact insulin sensitivity. However, it is not clear if changes occur as an early event in response to overfeeding and insulin resistance, or as a later consequence of the obese state. In this study, we show that overfeeding resulted in weight gain, increased lipid deposition in adipose tissue and liver and reduced insulin sensitivity. In parallel, increases in metabolically protective cytokines adiponectin and FGF21 were observed. No changes were detected in the metabolically adverse cytokines FABP4, PAI-1 and lipocalin-2, suggesting that these may be altered as a later consequence of the obese state. 

Adiponectin is primarily produced by the adipocyte and acts as a metabolically protective cytokine, with beneficial actions not only on insulin sensitivity, but also on the cardiovascular system [4]. Circulating adiponectin concentrations are not changed in response to a single high fat meal [24]. However, weight loss significantly increases circulating adiponectin [25]. Here, we observed that total adiponectin levels were increased in response to short-term overfeeding and weight gain. This is also previously reported after 5 days of high fat overfeeding in lean young men [26], thus we confirm and extend these findings to show elevations are sustained at least to 28 days in healthy humans. Moreover, we observed that the increase in adiponectin was correlated with the amount of weight gained during overfeeding. Whilst this response appears counterintuitive, since insulin sensitivity was reduced, it may be the metabolically healthy response in an attempt to maintain an anti-inflammatory profile and glucose homeostasis in liver and muscle. However, it should be noted that adiponectin circulates in multimeric forms [4], with the high molecular weight (HMW) being the biologically active form, which was not assessed in this study.

FGF21 is also postulated to be a metabolically protective cytokine since treatment with FGF21 mimetics protects against obesity, fatty liver and insulin resistance in mouse and primates [3,14]. Weight loss does not alter FGF21 levels in obese humans [27], but FGF21 is increased in response to 24-hours of fasting in mice and stimulates adipose tissue lipolysis and lipid metabolism [28]. In humans, fasting for between 1-3 days did not alter FGF21 [29,30], but fasting for 7 days significantly increased FGF21 [31]. FGF21 was also increased by lipid-heparin infusion which concurrently increased NEFA above 1.5 mmol/L [32]. These studies suggest that under conditions of increased delivery of fatty acids to the liver, FGF21 is increased and promotes lipid oxidation. Supporting this concept, high fat liquid gavage also increased expression of FGF21 in mouse liver [33]. In this study, we observed a transient increase in circulating FGF21 after 3-days of overfeeding and a similar finding has been reported in healthy men after 5 days of overfeeding [34]. This supports the notion that FGF21 is increased by conditions that likely promoted increased delivery of fatty acids to the liver. However, we do not have evidence of a role for FGF21 in promoting lipid oxidation under these conditions. Rather, FGF21 was increased alongside an increase in glucose oxidation at day 3 and returned to baseline despite an increase in measured lipid content in the liver at day 28. Additionally, we also observed a positive relationship between FGF21 and respiratory quotient at baseline. Whilst this is based on correlational analysis only, there is some evidence to support the concept that FGF21 may be regulated by both fasting and feeding signals, since the promoter region not only has a peroxisome proliferator activated receptor-α (PPARα) element that responds to fatty acids but a carbohydrate response element (ChREBP) that responds to glucose [35]. Of note however, FGF21 is also increased by changes in glucagon [36], which are known to be increased by overfeeding in humans [37]. Further study into the role of FGF21 under conditions of energy excess in humans is required. 

Serum levels of FABP4 are elevated in obesity and metabolic syndrome and may contribute to development of nonalcoholic steatohepatitis (NASH) [38]. We also observed relationships between serum FABP4 and numerous measures of adiposity and insulin resistance at baseline. However, FABP4 was not changed in response to overfeeding and moderate weight gain, suggesting that this is not nutritionally regulated or responsive to acute gains in liver fat. Our results are in contrast to previous studies that have reported that moderate exercise and weight losses produce small reductions in FABP4 [39], but are in line with reports in mouse that show elevations in FABP4 are aligned with models of NASH rather than fatty liver per se [33]. Lipocalin-2 is elevated in animal models of obesity and in obese humans [11]. However, the results from studies into the phenotype of the lipocalin-2 knockout mouse are mixed, with reports of no phenotype, as well as protection from and promotion of an obesity phenotype and insulin resistance [40-42]. Weight loss may reduce lipocalin-2 in humans [43], however this is not observed consistently [39]. In this study, we did not observe any relationship between lipocalin-2 and adiposity or insulin resistance at baseline. Similarly, we did not observe any relationships between obesity and PAI-1. Both may simply reflect the narrow BMI band investigated. However, we also observed no change in PAI-1 or lipocalin-2 in response to overfeeding and insulin resistance. In this cohort, short term overfeeding did not alter macrophage infiltration of adipose tissue [17], and therefore we suggest that PAI-1 and lipocalin-2 may be elevated as a consequence of macrophage infiltration of adipose tissue in the obese state [1]. 

In summary, over-nutrition increased body weight and markers of insulin resistance in healthy humans. Serum levels of adiponectin and FGF-21 were increased by overfeeding, possibly in an attempt to maintain insulin sensitivity. PAI-I, FABP4 and lipocalin 2 were not altered by overfeeding and moderate weight gain suggesting that changes in these cytokines are a later consequence of the obese state. 

## References

[1] Heilbronn LK, Campbell LV (2008) Adipose tissue macrophages, low grade inflammation and insulin resistance in human obesity. Curr Pharm Des 14: 1225-1230. doi:10.2174/138161208784246153. PubMed: 18473870. 18473870

[2] Kotronen A, Yki-Järvinen H (2008) Fatty liver: a novel component of the metabolic syndrome. Arterioscler Thromb Vasc Biol 28: 27-38. PubMed: 17690317. 17690317 10.1161/ATVBAHA.107.147538

[3] Kharitonenkov A, Shiyanova TL, Koester A, Ford AM, Micanovic R et al. (2005) FGF-21 as a novel metabolic regulator. J Clin Invest 115: 1627-1635. doi:10.1172/JCI23606. PubMed: 15902306. 15902306 PMC1088017

[4] Hickman IJ, Whitehead JP (2012) Structure, signalling and physiologic role of adiponectin-dietary and exercise- related variations. Curr Med Chem 19: 5427-5443. doi:10.2174/092986712803833155. PubMed: 22876920. 22876920

[5] Makowski L, Hotamisligil GS (2005) The role of fatty acid binding proteins in metabolic syndrome and atherosclerosis. Curr Opin Lipidol 16: 543-548. doi:10.1097/01.mol.0000180166.08196.07. PubMed: 16148539. 16148539 PMC3904771

[6] Xu A, Wang Y, Xu JY, Stejskal D, Tam S et al. (2006) Adipocyte fatty acid-binding protein is a plasma biomarker closely associated with obesity and metabolic syndrome. Clin Chem 52: 405-413. doi:10.1373/clinchem.2005.062463. PubMed: 16423904. 16423904

[7] Milner KL, van der Poorten D, Xu A, Bugianesi E, Kench JG et al. (2009) Adipocyte fatty acid binding protein levels relate to inflammation and fibrosis in nonalcoholic fatty liver disease. Hepatology 49: 1926-1934. doi:10.1002/hep.22896. PubMed: 19475694. 19475694

[8] Xu A, Tso AW, Cheung BM, Wang Y, Wat NM et al. (2007) Circulating adipocyte-fatty acid binding protein levels predict the development of the metabolic syndrome: a 5-year prospective study. Circulation 115: 1537-1543. doi:10.1161/CIRCULATIONAHA.106.647503. PubMed: 17389279. 17389279

[9] Maeda K, Cao H, Kono K, Gorgun CZ, Furuhashi M et al. (2005) Adipocyte/macrophage fatty acid binding proteins control integrated metabolic responses in obesity and diabetes. Cell Metab 1: 107-119. doi:10.1016/j.cmet.2004.12.008. PubMed: 16054052. 16054052

[10] Flo TH, Smith KD, Sato S, Rodriguez DJ, Holmes MA et al. (2004) Lipocalin 2 mediates an innate immune response to bacterial infection by sequestrating iron. Nature 432: 917-921. doi:10.1038/nature03104. PubMed: 15531878. 15531878

[11] Wang Y, Lam KS, Kraegen EW, Sweeney G, Zhang J et al. (2007) Lipocalin-2 is an inflammatory marker closely associated with obesity, insulin resistance, and hyperglycemia in humans. Clin Chem 53: 34-41. PubMed: 17040956. 17040956 10.1373/clinchem.2006.075614

[12] Alessi M-C, Juhan-Vague I (2006) PAI-1 and the Metabolic Syndrome: Links, Causes, and Consequences. Arterioscler Thromb Vasc Biol 26: 2200-2207. doi:10.1161/01.ATV.0000242905.41404.68. PubMed: 16931789. 16931789

[13] Schneider DJ, Sobel BE (2012) PAI-1 and Diabetes: A Journey From the Bench to the Bedside. Diabetes Care 35: 1961-1967. doi:10.2337/dc12-0638. PubMed: 22996180. 22996180 PMC3447837

[14] Foltz IN, Hu S, King C, Wu X, Yang C et al. (2012) Treating Diabetes and Obesity with an FGF21-Mimetic Antibody Activating the βKlotho/FGFR1c Receptor Complex. Sci Transl Med 4: 162ra153 PubMed: 23197570. 10.1126/scitranslmed.3004690 23197570

[15] Zhang X, Yeung DCY, Karpisek M, Stejskal D, Zhou Z-G et al. (2008) Serum FGF21 Levels Are Increased in Obesity and Are Independently Associated With the Metabolic Syndrome in Humans. Diabetes 57: 1246-1253. doi:10.2337/db07-1476. PubMed: 18252893. 18252893

[16] Fisher FM, Chui PC, Antonellis PJ, Bina HA, Kharitonenkov A et al. (2010) Obesity is a fibroblast growth factor 21 (FGF21)-resistant state. Diabetes 59: 2781-2789. doi:10.2337/db10-0193. PubMed: 20682689. 20682689 PMC2963536

[17] Tam CS, Viardot A, Clément K, Tordjman J, Tonks K et al. (2010) Short-Term Overfeeding May Induce Peripheral Insulin Resistance Without Altering Subcutaneous Adipose Tissue Macrophages in Humans. Diabetes 59: 2164-2170. doi:10.2337/db10-0162. PubMed: 20547978. 20547978 PMC2927938

[18] Samocha-Bonet D, Campbell LV, Viardot A, Freund J, Tam CS et al. (2010) A family history of type 2 diabetes increases risk factors associated with overfeeding. Diabetologia 53: 1700-1708. doi:10.1007/s00125-010-1768-y. PubMed: 20461357. 20461357

[19] Samocha-Bonet D, Campbell LV, Mori TA, Croft KD, Greenfield JR et al. (2012) Overfeeding reduces insulin sensitivity and increases oxidative stress, without altering markers of mitochondrial content and function in humans. PLOS ONE 7: e36320. doi:10.1371/journal.pone.0036320. PubMed: 22586466. 22586466 PMC3346759

[20] Ong KL, Rye K-A, O'Connell R, Jenkins AJ, Brown C et al. (2012) Long-Term Fenofibrate Therapy Increases Fibroblast Growth Factor 21 and Retinol-Binding Protein 4 in Subjects with Type 2 Diabetes. J Clin Endocrinol Metab 97: 4701-4708. doi:10.1210/jc.2012-2267. PubMed: 23144467. 23144467

[21] Xiao Y, Xu A, Hui X, Zhou P, Li X et al. (2013) Circulating Lipocalin-2 and Retinol-Binding Protein 4 Are Associated with Intima-Media Thickness and Subclinical Atherosclerosis in Patients with Type 2 Diabetes. PLOS ONE 8: e66607. doi:10.1371/journal.pone.0066607. PubMed: 23799122. 23799122 PMC3684582

[22] Yu H, Xia F, Lam KSL, Wang Y, Bao Y et al. (2011) Circadian Rhythm of Circulating Fibroblast Growth Factor 21 Is Related to Diurnal Changes in Fatty Acids in Humans. Clin Chem 57: 691-700. doi:10.1373/clinchem.2010.155184. PubMed: 21325103. 21325103

[23] Li H, Dong K, Fang Q, Hou X, Zhou M et al. (2013) High serum level of fibroblast growth factor 21 is an independent predictor of non-alcoholic fatty liver disease: A 3-year prospective study in China. J Hepatol 58: 557-563. doi:10.1016/S0168-8278(13)61386-0. PubMed: 23142063. 23142063

[24] Peake PW, Kriketos AD, Denyer GS, Campbell LV, Charlesworth JA (2003) The postprandial response of adiponectin to a high-fat meal in normal and insulin-resistant subjects. Int J Obes 27: 657-662. doi:10.1038/sj.ijo.0802289. PubMed: 12833108. 12833108

[25] Pasarica M, Tchoukalova YD, Heilbronn LK, Fang X, Albu JB et al. (2009) Differential effect of weight loss on adipocyte size subfractions in patients with type 2 diabetes. Obesity (Silver Spring) 17: 1976-1978. doi:10.1038/oby.2009.219. PubMed: 19629054. 19629054 PMC3985333

[26] Brøns C, Jensen CB, Storgaard H, Hiscock NJ, White A et al. (2009) Impact of short-term high-fat feeding on glucose and insulin metabolism in young healthy men. J Physiol 587: 2387-2397. doi:10.1113/jphysiol.2009.169078. PubMed: 19332493. 19332493 PMC2697306

[27] Mai K, Schwarz F, Bobbert T, Andres J, Assmann A et al. (2011) Relation between fibroblast growth factor-21, adiposity, metabolism, and weight reduction. Metabolism 60: 306-311. doi:10.1016/j.metabol.2010.02.016. PubMed: 20362303. 20362303

[28] Badman MK, Pissios P, Kennedy AR, Koukos G, Flier JS et al. (2007) Hepatic Fibroblast Growth Factor 21 Is Regulated by PPARα and Is a Key Mediator of Hepatic Lipid Metabolism in Ketotic States. Cell Metab 5: 426-437. doi:10.1016/j.cmet.2007.05.002. PubMed: 17550778. 17550778

[29] Andersen B, Beck-Nielsen H, Højlund K (2011) Plasma FGF21 displays a circadian rhythm during a 72-h fast in healthy female volunteers. Clin Endocrinol 75: 514-519. doi:10.1111/j.1365-2265.2011.04084.x. PubMed: 21521350. 21521350

[30] Dushay J, Chui PC, Gopalakrishnan GS, Varela-Rey M, Crawley M et al. (2010) Increased fibroblast growth factor 21 in obesity and nonalcoholic fatty liver disease. Gastroenterology 139: 456-463. doi:10.1053/j.gastro.2010.04.054. PubMed: 20451522. 20451522 PMC4862867

[31] Gälman C, Lundåsen T, Kharitonenkov A, Bina HA, Eriksson M et al. (2008) The Circulating Metabolic Regulator FGF21 Is Induced by Prolonged Fasting and PPAR± Activation in Man. Cell Metab 8: 169-174. doi:10.1016/j.cmet.2008.06.014. PubMed: 18680716. 18680716

[32] Mai K, Bobbert T, Groth C, Assmann A, Meinus S et al. (2010) Physiological modulation of circulating FGF21: relevance of free fatty acids and insulin. Am J Physiol Endocrinol Metab 299: E126-E130. doi:10.1152/ajpendo.00020.2010. PubMed: 20424140. 20424140

[33] Gaemers IC, Stallen JM, Kunne C, Wallner C, van Werven J et al. (2011) Lipotoxicity and steatohepatitis in an overfed mouse model for non-alcoholic fatty liver disease. Biochim Biophys Acta 1812: 447-458. doi:10.1016/j.bbadis.2011.01.003. PubMed: 21216282. 21216282

[34] Vienberg SG, Brøns C, Nilsson E, Astrup A, Vaag A et al. (2012) Impact of short-term high-fat feeding and insulin-stimulated FGF21 levels in subjects with low birth weight and controls. Eur J Endocrinol 167: 49-57. doi:10.1530/EJE-12-0039. PubMed: 22529197. 22529197

[35] Uebanso T, Taketani Y, Yamamoto H, Amo K, Ominami H et al. (2011) Paradoxical Regulation of Human FGF21 by Both Fasting and Feeding Signals: Is FGF21 a Nutritional Adaptation Factor? PLOS ONE 6: e22976. doi:10.1371/journal.pone.0022976. PubMed: 21829679. 21829679 PMC3148241

[36] Habegger KM, Stemmer K, Cheng C, Müller TD, Heppner KM et al. (2013) Fibroblast Growth Factor 21 Mediates Specific Glucagon Actions. Diabetes 62: 1453-1463. doi:10.2337/db12-1116. PubMed: 23305646. 23305646 PMC3636653

[37] Oppert J-M, Nadeau A, Tremblay A, Després J-P, Thériault G et al. (1995) Plasma glucose, insulin, and glucagon before and after long-term overfeeding in identical twins. Metabolism 44: 96-105. doi:10.1016/0026-0495(95)90295-3. PubMed: 7854173. 7854173

[38] Yoon MY, Sung JM, Song CS, Lee WY, Rhee EJ et al. (2012) Enhanced A-FABP expression in visceral fat: potential contributor to the progression of NASH. Clin Mol Hepatol 18: 279-286. doi:10.3350/cmh.2012.18.3.279. PubMed: 23091808. 23091808 PMC3467431

[39] Choi KM, Kim TN, Yoo HJ, Lee KW, Cho GJ et al. (2009) Effect of exercise training on A-FABP, lipocalin-2 and RBP4 levels in obese women. Clin Endocrinol 70: 569-574. doi:10.1111/j.1365-2265.2008.03374.x. PubMed: 18710473. 18710473

[40] Jun LS, Siddall CP, Rosen ED (2011) A minor role for lipocalin 2 in high-fat diet-induced glucose intolerance. Am J Physiol Endocrinol Metab 301: E825-E835. doi:10.1152/ajpendo.00147.2011. PubMed: 21771968. 21771968 PMC4073944

[41] Guo H, Jin D, Zhang Y, Wright W, Bazuine M et al. (2010) Lipocalin-2 Deficiency Impairs Thermogenesis and Potentiates Diet-Induced Insulin Resistance in Mice. Diabetes 59: 1376-1385. doi:10.2337/db09-1735. PubMed: 20332347. 20332347 PMC2874698

[42] Law IKM, Xu A, Lam KSL, Berger T, Mak TW et al. (2010) Lipocalin-2 Deficiency Attenuates Insulin Resistance Associated With Aging and Obesity. Diabetes 59: 872-882. doi:10.2337/db09-1541. PubMed: 20068130. 20068130 PMC2844835

[43] Koiou E, Tziomalos K, Katsikis I, Kandaraki EA, Kalaitzakis E et al. (2012) Weight loss significantly reduces serum lipocalin-2 levels in overweight and obese women with polycystic ovary syndrome. Gynecol Endocrinol 28: 20-24. doi:10.3109/09513590.2011.588745. PubMed: 21756081. 21756081

